# Clinical Lecture on Bed-Sores

**Published:** 1873-07

**Authors:** James Paget

**Affiliations:** Lecturer on Clinical Surgery at St. Bartholomew’s Hospital


*Clinical Lecture on Bed-Sores.* By SIR JAMES PAGET, F.R.S., etc., Lecturer on Clinical Surgery at St. Bartholomew's Hospital.

Bed-sores may be defined as the sloughing and mortification or death of a part produced by pressure. When we press on any part of our bodies for a moment, on the removal of the pressure the part is quite white, owing to the blood having been pressed out. The color immediately returns, however. In bed-sores, the pressure is continual, the blood is driven away, nourishment ceases, and death of the part takes place. There are three different forerunners of bed-sores. 1. Inflammation; the prominent parts, *e.g.*, the sacrum, posterior superior spine of the ilium, the trochanters, and the ends of the spines of the vertebræ, are seen to be red. 2. They may be simply pale and white. 3. They may be purple or yellow, from the extravasation of blood or bloody fluid. Sloughing follows these in the skin and subcutaneous tissue and fat. These latter die before the skin, sloughing proceeds faster in them, and so when the skin comes away, the place formerly occupied by these tissues is empty. Then the deeper parts die—muscles, bone, until sometimes the spinal cord itself is exposed. Now, bed-sores occur in those who are absolutely at rest. If there is the slightest movement from one side to the other, bed-sores may be averted. A man with simple fracture of the femur, previously healthy, can move himself slightly from side to side, and does so instinctively.

No man with simple fracture of femur ought to rise from his bed with a bed-sore. It would be the consequence of gross neglect if he did. In the case of those whose lower limbs are paralyzed, there can be no motion whatever, and so they are liable to bed-sores.

The time when bed-sores begin to make their appearance is about fourteen days — that is, in the case of a healthy man who is absolutely unmoved. They will, of course, be accelerated by dirt, if his urine and fæces are not constantly removed. There are certain cases which are specially favorable for bed-sores: the old, especially those with fractured neck of femur, and those that are the fattest and heaviest, and most likely to become œdematous. Among ordinary persons, those that are the thinnest. When, as is commonly said, their bones are ready to start through their skin, the amount of tissues between the skin and projecting point of bone is so small that it soon, as it were, wears away, and bed-sores ensue. Those again in a state of fever, such as the lowest kinds of typhus, can scarcely by any means be saved from them. Their whole system is so poor and degenerated that sloughing takes place without any pressure at all; and you may see the ends of the nose, ears, etc., sloughing from the bad supply of blood. Continuous hectic fever is a state in which they appear, being an exception to the general class of consumptive patients, who, though they may lie in bed for months, rarely have bed-sores. They manage to move slightly and thus avert them. Pyæmia is another source, and is illustrated by a case in the Hospital: a man who was admitted with phlegmonous erysipelas of a limb and was treated for it. On account of some misconduct he was discharged: after a while he came back with pyæmia and an enormous bed-sore. His skin is very pallid and soft, and does not properly discharge its functions, and there is every reason to believe that every other organ of his body is in a similar state. His lungs may be auscultated and his urine examined, and nothing at all found wrong with them, and yet I venture to state that neither the lungs nor kidneys are performing their functions as they ought. A pyæmic subject, being so ill-nourished, is especially liable to bed-sores. Intense fever is also a productive agent. The man whose thigh was amputated a short time since, had a most acute and intense attack of fever, and large bed-sores appeared. Now the fever is gone, the local disease is removed, and the bed-sores are healing very rapidly. The risk of bed-sores in the old with fractured neck of femur is chiefly in the first week, therefore treatment with a view to preventing them should commence immediately the patient takes to bed. After the first week the risk is not nearly so great. There is one peculiar class in which bed-sores rapidly appear, and that is rapid destruction with inflammation of spinal marrow. If in a fracture of the spine, a portion of the spinal cord below the seat of fracture be irritated and inflamed, sloughing will ensue in those parts to which the nerves given off below the irritated part proceed. And this will take place in two or three days. Sir B. Brodie mentions a case in which a large slough formed on the heel in twenty-four hours. No doubt there were other causes for this. Two or three days is the usual time. The same takes place in diseases of the spinal cord, especially in acute pyelitis. There is not so much risk of sloughing in parts deprived of nerve force as in parts whose nerve force is irritated and disturbed.

Now let us look at the means of preventing bed-sores, for nine-tenths of your care must be devoted to this; for if once they appear, it is very difficult to get rid of them.

First of all, look to the bed. Good bed-making is an indispensable thing in the prevention of bed-sores. Several beds have been made especially for this purpose, of which the best is Dr. Arnott's. It consists of a chest full of water; on the top of this is a water-proof sheet, and over this an ordinary sheet on which the patient is laid. Here the patient is absolutely floating on water. The waterproof sheet is not drawn tight, but adapts itself to every part of the patient. A patient might lie on this for years and never have a bed-sore. Inferior to this, but very good, is Hooper's bed. Here the waterproof on the bed is tight. They will avert bed-sores for a long time, but I should not like to say that a patient would never get a bed-sore on them. But you cannot have these everywhere; you cannot take them about to every one who may need them, and there are many cases in which they cannot be used at all, as in cases of fractured neck of femur, acute inflammation of knee-joint, and many others.

In ordinary beds the best thing is an ordinary firm mattress of horse-hair; and it must rest on boards. Cords are the worst possible things, as after twenty-four hours or so they give under the weight of the patient, and the most prominent parts are pressed upon. Iron gives after two or three weeks. Not so boards. It must be quite level. Under the horse-hair it is better, if possible, to have a spring or straw mattress. Feather-beds and the like are, of course, to be utterly condemned. If possible, have two beds, so that you may lift the patient into the other when it wants making. You thus avoid making beds under him.

The next thing is to harden the skin. The best application for this is a solution of one part of nitrous ether in three of water. If the back is frequently washed with this, bed-sores may be completely averted. There is in the Hospital a man paralyzed in his lower limbs; he has been in this state for ten months. By the good nursing of the sister of his ward bed-sores have been keep away. This application of nitrous ether has been used: solution of one grain of perchloride of mercury, with two drachms of nitrous ether, and six ounces of water, is another good thing. Whisky is used in Scotland, as is brandy sometimes in England, but these are not so good. In Germany they use a solution of tannic acid. When the parts look as if they were going to slough, these spirit applications may be too strong, and then a solution of gutta percha in chloroform is very useful. Next we have to prevent pressure on those parts where bed-sores are likely to occur. These are the middle line of the sacrum, after that, in thin persons, the posterior superior spines of the ilium and the sacro-iliac articulations, then the trochanters of the femur. The chief thing is a frequent change of posture. If a patient can lie in four different positions during the day, bed-sores may be prevented. He may lie on his back, each side, and on his face. Of course, you could not make a stout person lie on his face; he would simply suffocate. This change prevents the gravitation of the blood. This may easily be seen by looking at the back of a subject in the post-mortem room. The back is quite red from this cause.

When patients lie on their backs they may be saved for a time by dividing a mattress and leaving a space of six inches between the halves. You may thus save the sacrum, which will have no pressure on it. The case before referred to was treated so, but sores came on the ilium and trochanters.

Large cushions made of amadou in the shape of a horse shoe are very good. Isinglass plaster or felt water-pillows and pads of cotton-wool may also be used with advantage. In speaking of the mode of curing bed-sores, already formed, let me remind you to continue your preventive treatment just as if there were none, lest they come in other parts.

During the sloughing there is nothing better than a poultice of equal parts of linseed and bread, and enough charcoal to have a deodorizing effect. Carrot and turnip poultices are also deodorizing, but they are not so good as the first. The poultice is best spread on ordinary tow. When spread on linen, etc., folds are liable to form, and if the patient is on these they promote the bed-sore. When slough begins to separate, the resin or other stimulating ointment should be spread on the surface of the poultice.

When the slough has separated, the sore should be dressed with resin ointment or Peruvian balsam, or equal parts of these, in the following manner: little bits of cotton wool should be slightly spread with the ointment, and put into the sore until it is quite full. They thus make an equable soft surface for the sore. These are the chief local means for curing bed-sores. As regards internal treatment, do not stimulate. Let the diet be gentle but good; plenty of milk and bread; little or no meat, and a small quantity of wine.—*The Students' Journal.*

